# Impact of catheter-induced iatrogenic coronary artery dissection with or without postprocedural flow impairment: A report from a Japanese multicenter percutaneous coronary intervention registry

**DOI:** 10.1371/journal.pone.0204333

**Published:** 2018-09-28

**Authors:** Takahiro Hiraide, Mitsuaki Sawano, Yasuyuki Shiraishi, Ikuko Ueda, Yohei Numasawa, Shigetaka Noma, Kouji Negishi, Takahiro Ohki, Shinsuke Yuasa, Kentaro Hayashida, Hiroaki Miyata, Keiichi Fukuda, Shun Kohsaka

**Affiliations:** 1 Department of Cardiology, Keio University School of Medicine, Tokyo, Japan; 2 Department of Cardiology, Japanese Red Cross Ashikaga Hospital, Tochigi, Japan; 3 Department of Cardiology, Saiseikai Utsunomiya Hospital, Tochigi, Japan; 4 Department of Cardiology, Yokohama Municipal Citizens’ Hospital, Kanagawa, Japan; 5 Department of Cardiology, Tokyo Dental College Ichikawa General Hospital, Chiba, Japan; 6 Department of Healthcare Quality Assessment Graduate School of Medicine, The University of Tokyo, Tokyo, Japan; Osaka University Graduate School of Medicine, JAPAN

## Abstract

Despite the ever-increasing complexity of percutaneous coronary intervention (PCI), the incidence, predictors, and in-hospital outcomes of catheter-induced coronary artery dissection (CICAD) is not well defined. In addition, there are little data on whether persistent coronary flow impairment after CICAD will affect clinical outcomes. We evaluated 17,225 patients from 15 participating hospitals within the Japanese PCI registry from January 2008 to March 2016. Associations between CICAD and in-hospital adverse cardiovascular events were evaluated using multivariate logistic regression. Outcomes of patients with CICAD with or without postprocedural flow impairment (TIMI flow ≤ 2 or 3, respectively) were analyzed. The population was predominantly male (79.4%; mean age, 68.2 ± 11.0 years); 35.6% underwent PCI for complex lesions (eg. chronic total occlusion or a bifurcation lesion.). CICAD occurred in 185 (1.1%), and its incidence gradually decreased (p < 0.001 for trend); postprocedural flow impairment was observed in 43 (23.2%). Female sex, complex PCI, and target lesion in proximal vessel were independent predictors (odds ratio [OR], 2.18; 95% confidence interval [CI], 1.53–3.10; OR, 2.19; 95% CI, 1.58–3.04; and OR, 1.55; 95% CI, 1.06–2.28, respectively). CICAD was associated with an increased risk of in-hospital adverse events (composite of new-onset cardiogenic shock and new-onset heart failure) regardless of postprocedural flow impairment (OR, 10.9; 95% CI, 5.30–22.6 and OR, 2.27; 95% CI, 1.20–4.27, respectively for flow-impaired and flow-recovered CICAD). In conclusion, CICAD occurred in roughly 1% of PCI cases; female sex, complex PCI, and proximal lesion were its independent risk factors. CICAD was associated with adverse in-hospital cardiovascular events regardless of final flow status. Our data implied that the appropriate selection of PCI was necessary for women with complex lesions.

## Introduction

In the pre-stent era, catheter-induced coronary artery dissection (CICAD) occurred in approximately 30% of cases during percutaneous coronary intervention (PCI) [1–3]. With the arrival of bare metal and drug-eluting stents (DES), the incidence of CICAD decreased down to 2–3% in the early 2000s [4], with a majority of the cases achieving normal distal flow at final angiography owing to the advancement of various bailout techniques [5,6]. Naturally, this advancement has led more interventionists to perform PCI in patients with more high-risk anatomic features, such as chronic total occlusion (CTO), bifurcation, and left main trunk lesions [7]. Consequently, procedural complications such as abrupt closure, perforation, device embolization, and CICAD have re-emerged and are under the spotlight in the contemporary PCI era [8,9]. Although there were several previously published series, their small size has limited the robustness of any conclusion; in particular, the impact of flow-limiting vs flow-recovered CICAD has not yet been defined.

The aim of this observational multicenter study was to assess the incidence, predictors, and in-hospital outcomes of CICAD. Specifically, we aimed to describe whether the impact of CICAD would differ by postprocedural coronary flow impairment status. We hypothesized that the occurrence of CICAD may be related to a worse in-hospital outcome, more so in CICAD patients with coronary flow impairment (Thrombolysis in Myocardial Infarction [TIMI] flow 0–2).

## Materials and methods

### Study population

We evaluated 17,225 patients from 15 participating hospitals within the Japanese Cardiovascular Database Keio interhospital Cardiovascular Studies (JCD-KiCS) registry from January 2008 to March 2016. The prospective JCD-KiCS registry was designed to collect clinical variables and outcomes data of consecutive PCI patients with dedicated clinical research coordinators assigned to each site. Approximately 200 variables under the appropriate nomenclature coding were collected from each patient. The JCD-KiCS registry includes 15 institutions within the metropolitan Tokyo area, consisting of mostly large tertiary care referral centers. The average annual PCI-volume was 228 between 2009 and 2013 across 15 hospitals. Between 2013 and 2016, we concentrated our data collection resources to four high-volume centers with a PCI volume of over 400 cases/year contributing to 6,465 cases within this current database. The participating hospitals were instructed to record and register data from consecutive hospital visits for PCI using an internet-based data collection system. Patient demographics, coronary lesion characteristics, and post-procedural complications were recorded. All PCI performed with any commercially available coronary devices were included.

The data entered were checked for completeness and internal consistency. Quality assurance of the data was achieved through automatic system validation and reporting of data completeness, education, and training by clinical research coordinators specifically trained for the present PCI registry. The senior study coordinator (I.U.) and exclusive on-site auditing by investigators (S.K. and H.M.) ensured the proper registration of each patient. The Institutional Review Board of Keio University School of Medicine as well as each participating hospital (Ashikaga Red Cross Hospital, Hino Municipal Hospital, Kawasaki Municipal Hospital, Eiju General Hospital, National Hospital Organization Saitama National Hospital, National Hospital Organization Tokyo Medical Center, Saiseikai Central Hospital, Saiseikai Utsunomiya Hospital, Saitama City Hospital, St Luke’s International Hospital, Tachikawa Hospital, Tokyo Dental College Ichikawa General Hospital, Yokohama Municipal Citizen’s Hospital, and Hiratsuka City Hospital (and Isehara Kyodo Hospital)) approved the JCD-KiCS registry study protocol. Verbal or written informed consent was routinely obtained from all patients before undergoing PCI.

### Definitions

Most of the demographic and angiographic definitions in the JCD-KiCS were created according to the National Cardiovascular Data Registry [10]. CICAD was defined as the appearance of contrast outside the expected luminal dimensions of the target coronary vessel that caused flow limitations (TIMI flow grade 0–2) of the distal vessels. Dissections with TIMI flow grade 3 (National Heart, Lung and Blood Institute [NHLBI] classification types A and B) were not recorded as complications since this type of dissection represented the “natural” response of the coronary wall to the mechanical injury of vessel stretching caused by high-pressure balloon inflation [11]. Spontaneous coronary artery dissections that were observed before the intervention were not considered CICAD. Patients who experienced CICAD were categorized into two groups according to their coronary flow recovery after procedure. Flow-impaired CICAD was identified when flow limitation was observed (TIMI flow grade ≤ 2) at the final angiography, whereas flow-recovered CICAD was identified when the distal flow was recovered to TIMI flow grade 3 at final angiography.

Complex PCI was defined as a procedure in which bifurcation or chronic total occlusion as the target lesion was observed [12,13]. Chronic total occlusion was indicated if the segment with 100% pre-procedure stenosis was presumed to be totally occluded for at least 3 months prior to the procedure. The proximal lesion was defined as segments 1 and 5 of the coronary artery according to American Heart Association classification [14]. Type C lesions were defined as diffuse (length > 2cm), excessive tortuosity of proximal segment, extremely angulated segments > 90 degrees, total occlusions > 3 months old and/or bridging collaterals, inability to protect major side branches, and degenerated vein grafts with friable lesions. Peripheral artery disease was indicated if the patients had arterial disease in the upper- or lower-extremity, renal, mesenteric, or abdominal aortic systems. Left ventricular ejection fraction was measured by contrast left ventriculography or echocardiography. Cardiogenic shock was defined as a sustained (>30 minutes) episode of systolic blood pressure < 90 mm Hg and/or cardiac index < 2.2 L/min/m^2^ determined to be secondary to cardiac dysfunction and/or the requirement for parenteral inotropic or vasopressor agents or mechanical support to maintain blood pressure and cardiac index above those specified levels [10]. Post-procedural myocardial infarction was defined if patients after PCI had elevated cardiac biomarkers, ischemic symptoms, or change of ECG.

### Study endpoints

The endpoints of this study were in-hospital all-cause mortality and in-hospital adverse cardiovascular events (i.e. new-onset cardiogenic shock, and new-onset heart failure) [12,15]. To assess the impact of flow limitation, the outcomes of patients with CICAD were analyzed separately according to postprocedural flow impairment. These endpoints were recorded in the database by the trained coordinators immediately after patient discharge. Heart failure is defined as physician documentation or report of any of the following clinical symptoms of heart failure: unusual dyspnea on light exertion; recurrent dyspnea occurring in the supine position; fluid retention, rales, jugular venous distension, or pulmonary edema on a physical exam; or pulmonary edema on a chest x-ray.

### Statistical analysis

**Fig 1:**
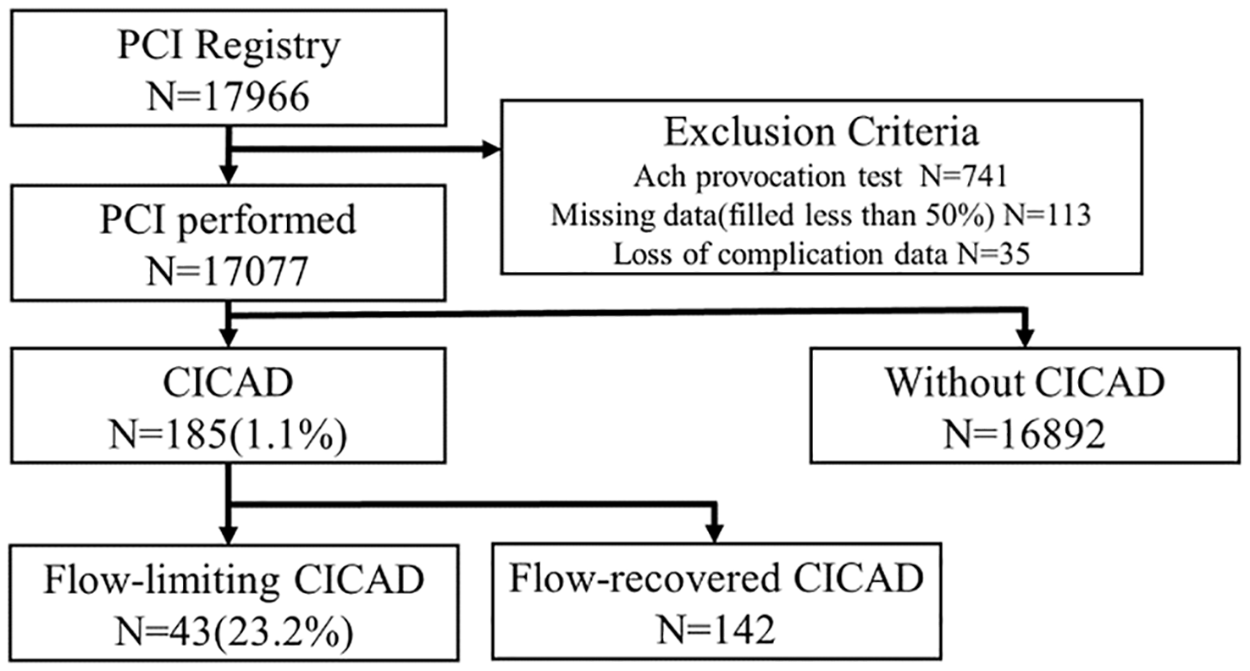
Study population flow diagram. CICAD was occurred in 1.1% of study population. CICAD = catheter-induced coronary artery dissection; PCI = percutaneous coronary intervention.

Continuous variables are expressed as median and (interquartile range). Categorical variables were presented as percentages. Patients with missing baseline data (n = 148; 0.8%) were excluded from the analysis. A total of 17,077 patients were divided into two groups by presence or absence of CICAD (Fig 1). Baseline characteristics were compared using the Mann-Whitney U test for continuous variables and the Chi-square or Fisher’s exact test for categorical variables. A trend analysis of the incidence of CICAD was performed using the Cochran-Armitage test. P values were two-sided with a significance level of 0.05. Multivariate logistic regression modeling was performed to determine the odds ratio (OR) of in-hospital mortality and in-hospital adverse cardiovascular events as well as the predictors of CICAD. Covariates on the multivariate analysis were selected from the clinically relevant pre-procedural patient and coronary lesion characteristics. As for the multivariate analysis on the predictors of CICAD, we chose the variables from clinically relevant pre-procedural characteristics (age, gender, BMI, diabetes, chronic kidney disease, use of hemodialysis, bifurcation and/or CTO lesion, proximal lesion, previous PCI, presentation with ACS). Known clinical variables associated with coronary arterial vulnerability has been described previously [16], and recent reports on CICAD [5, 11] have also demonstrated that technical variables such as lesion complexity (e.g., calcified lesions or ACC/AHA type B/C lesions), were associated with occurrence of CICAD. Since the post hoc selection of covariates would tend to lead to biased estimates of outcome [17], we included all of the above variables into our model. In the multivariate analysis on the in-hospital mortality, we adjusted for known predictors of adverse outcomes among patients undergoing PCI, including age, gender, presence of cardiogenic shock at admission, use of hemodialysis, and chronic lung disease [18, 19]. These selected variables are in line with variables included in the contemporary risk scores used in predicting the outcome of PCI [20]. Results are reported as OR with a 95% confidence interval (CI). Analyses were performed using SPSS software 24.0.1 (SPSS Inc., Chicago, IL, USA).

## Results

**Fig 2:**
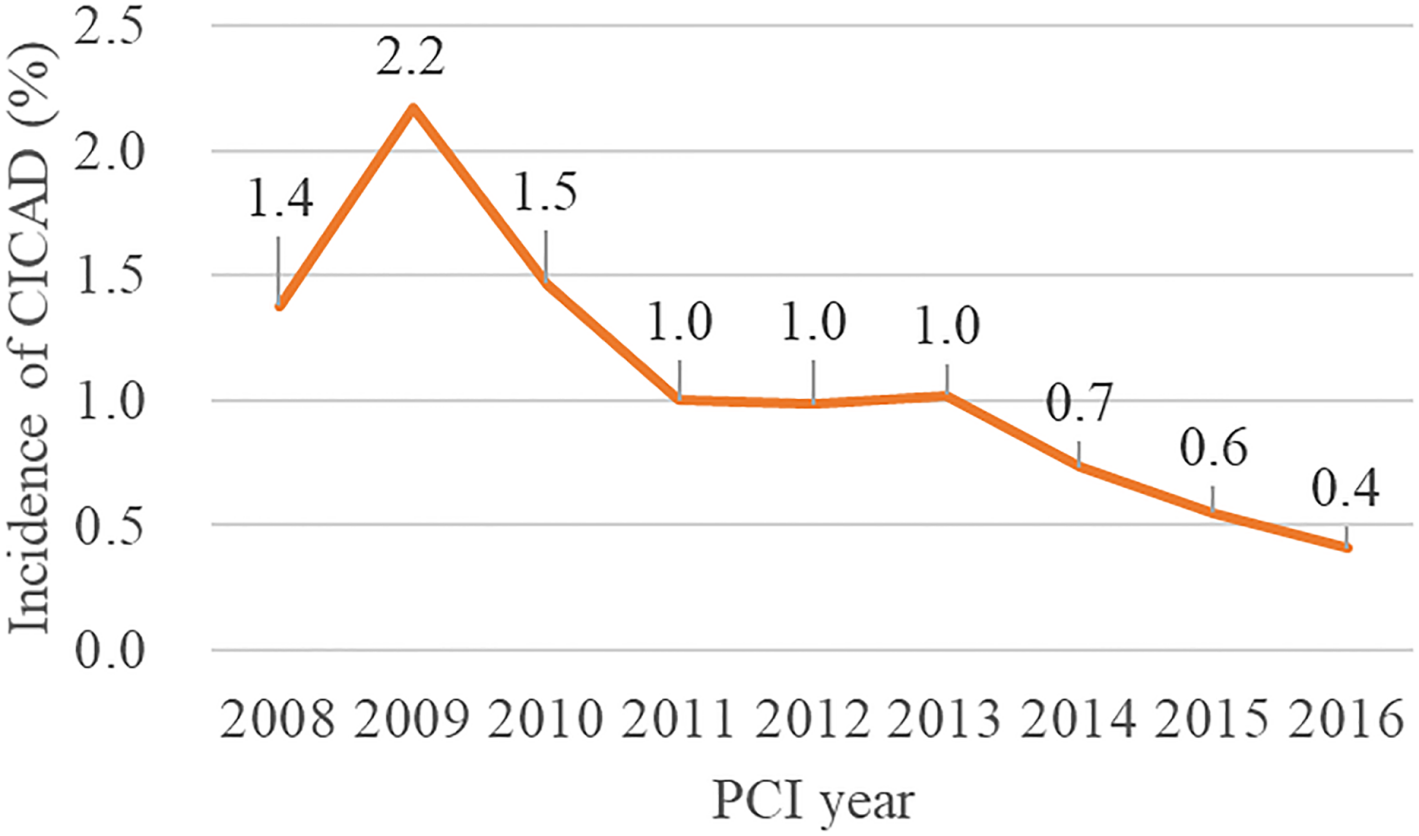
Annual incidence of catheter-induced coronary artery dissection. The annual incidence of CICAD was gradually decreasing in study period (p < 0.001 for trend with Cochran-Armitage test). CICAD = catheter-induced coronary artery dissection; PCI = percutaneous coronary intervention.

Between 2008 and 2016, CICAD occurred in 185 patients (1.1%) with the annual incidence gradually decreasing over time (p < 0.001 for trend) (Fig 2). CICAD was more frequently observed in women than in men (34.3% vs 20.5%, p < 0.001). Other baseline demographics, such as a history of myocardial infarction and left ventricular ejection fraction, were similar between the CICAD and non-CICAD groups. In terms of angiographical characteristics, CTO (16.2% vs 7.5%, p < 0.001), bifurcation (41.6% vs 30.2%, p = 0.001), proximal lesions (23.8% vs 17.3%, p = 0.025) and multi-vessel intervention (17.3% vs 10.2%, p = 0.002) were observed at a higher rate in patients with CICAD than without CICAD. Cardiogenic shock was observed in 7.6% (n = 11) of the patients in the CICAD group and 1.9% (n = 328) of those without CICAD. IABP was used in 20.9% of the patients in the CICAD group and 7.3% of non-CICAD group (p < 0.001). The use of percutaneous cardiopulmonary support (veno-arterial extracorporeal membrane oxygenation) was not significant between the two groups (1.6% vs 1.0%, p = 0.441). (Table 1).

**Table 1:** Baseline clinical and angiographic xharacteristics.

	With CICAD (N = 185)	Without CICAD (N = 16892)	P value
Age, y	69 (62, 76)	69 (61, 76)	0.419
Female sex	34.3	20.5	<0.001
BMI	23.4 (21.9, 26.3)	24.0 (21.9, 26.2)	0.566
Hypertension	69.8	74.6	0.146
Dyslipidemia	66.3	65.8	0.892
Diabetes mellitus	41.3	42.8	0.684
Smoking within 1 year	31.4	33.2	0.622
Family history of heart disease	9.5	11.7	0.373
CKD	48.2	43.9	0.255
Dialysis	4.1	4.7	0.685
Cerebral vascular disease	11.0	9.0	0.351
Peripheral artery disease	8.7	8.8	0.963
Previous MI	24.4	24.6	0.967
Previous PCI	5.8	5.2	0.754
Previous bypass surgery	37.0	38.6	0.658
Ejection fraction of LV	56.5 (45.5, 66.0)	60.0 (49.0, 68.0)	0.035
Acute coronary syndrome	41.6	44.8	0.414
Cardiogenic shock at admission	5.9	4.3	0.259
Cardiopulmonary arrest at admission	3.2	2.7	0.643
Lesion			
RCA	36.2	33.8	0.494
LMT	8.6	4.6	0.008
LAD	51.9	46.9	0.179
LCX	19.5	23.4	0.205
Proximal lesion	23.8	17.3	0.025
Bifurcation lesion	41.6	30.2	0.001
Type C lesion	47.6	33.9	<0.001
Chronic total occlusion	16.2	7.5	<0.001
Multi-vessel intervention	17.3	10.2	0.002
Target vessel stent length(mm)	20 (16, 28)	20 (16, 26)	0.222
Target vessel stent diameter(mm)	3.0 (2.5, 3.5)	3.0 (2.5, 3.5)	0.324
Intravascular ultrasound before PCI	34.3	39.1	0.216
Intra-aortic balloon pumping	20.9	7.3	<0.001
Pre-procedural use	15.8	24.5	0.001
Per-procedural use	52.6	64.2	
Post-procedural use	31.6	11.3	
Cardiogenic shock during PCI	7.6	1.9	<0.001
Percutaneous cardiopulmonary support (VA-ECMO)	1.6	1.0	0.441

Values are median (interquartile range) or n (%).

BMI = body mass index; CKD = chronic kidney disease; CICAD = catheter-induced coronary artery dissection; LAD = left anterior descending artery; LCX = left circumflex artery; LMT = left main trunk; LV = left ventricular; MI = myocardial infarction; PCI = percutaneous coronary intervention; RCA = right coronary artery; VA-ECMO = veno-arterial extracorporeal membrane oxygenation.

**Fig 3:**
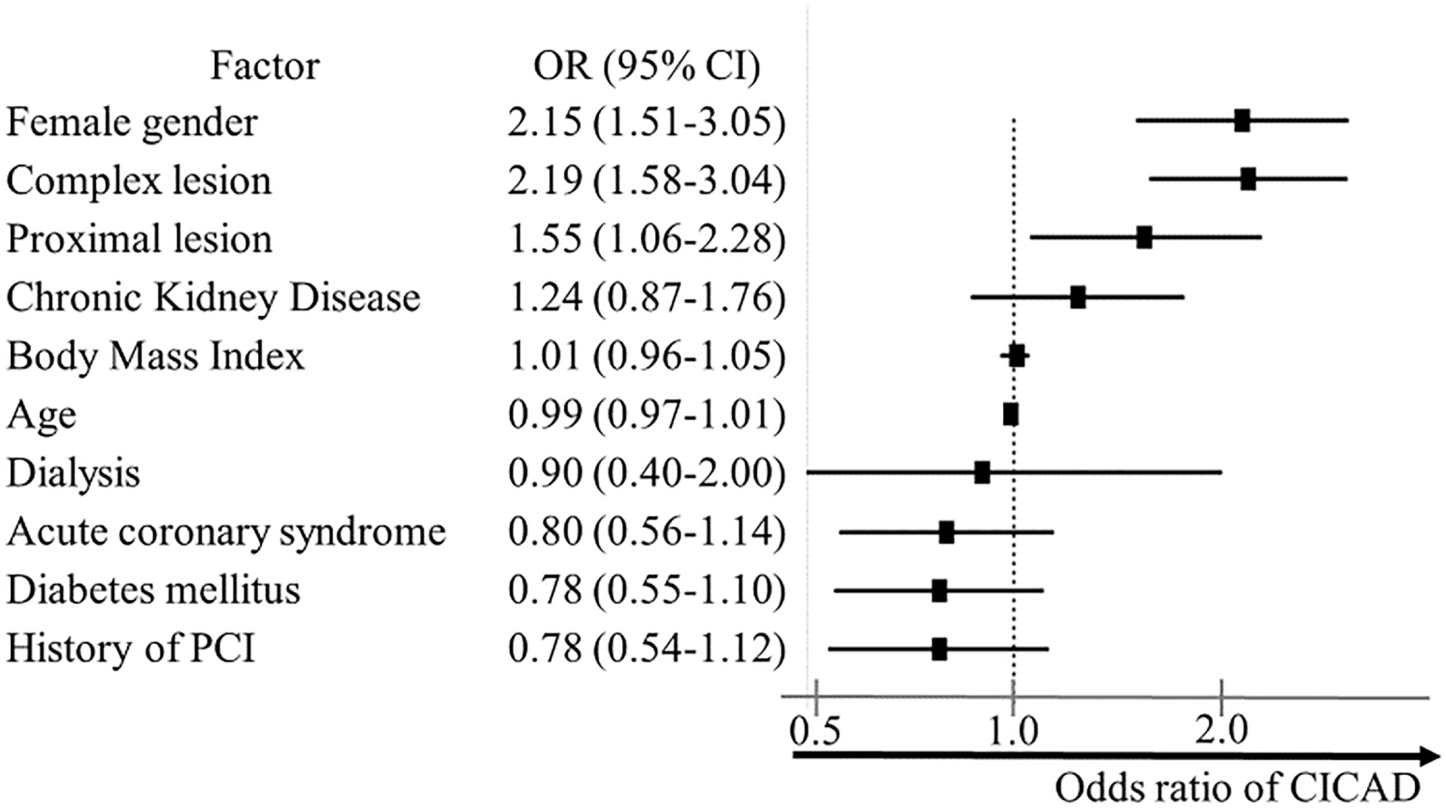
Predictors of catheter-induced coronary artery dissection. Multivariable predictors of iatrogenic coronary artery dissection (CICAD). Adjusted OR (point estimate) and 95% CI (error bars) indicate the likelihood ratio of CICAD from logistic regression. OR > 1 indicates increased odds of CICAD. CICAD = catheter-induced coronary artery dissection; OR = odds ratio; PCI = percutaneous coronary intervention.

Female sex (OR, 2.15; 95% CI, 1.51–3.05), complex lesion (OR, 2.19; 95% CI, 1.58–3.04), and proximal lesion PCI (OR, 1.55; 95% CI, 1.06–2.28) were independent risk factors of CICAD after the adjustment for known confounders (Fig 3). Women with complex PCI had an approximate four-fold increased risk of CICAD compared to men without complex lesion PCI (2.6% vs 0.6%, p < 0.001).

Overall, 416 patients (2.4%) died during hospitalization and 818 patients (4.8%) experienced in-hospital adverse cardiovascular events. CICAD was associated with significantly increased rates of in-hospital mortality (6.5% vs 2.4%, p = 0.002) as well as the in-hospital adverse cardiovascular events (14.1% vs 4.7%, p < 0.001). Both new onset of cardiogenic shock (7.6% vs 1.9%, p<0.001) and heart failure (5.4% vs 1.8%, p = 0.002) were higher in patients with CICAD. Post-procedural myocardial infarction was observed higher in CICAD group than in non-CICAD group (10.6% vs 1.6%, p < 0.001) (Table 2). After the adjustment for known confounders, CICAD was a significant predictor for in-hospital all-cause death (OR, 3.24; 95% CI, 1.63–6.44) and in-hospital adverse cardiovascular events (OR, 3.34; 95% CI, 2.06–5.42) (Table 3).

**Table 2:** Post-procedural complications and in-hospital outcomes.

	With CICAD (N = 185)	Without CICAD (N = 16892)	P value
In-hospital all-cause death	6.5	2.4	0.002
In-hospital adverse cardiovascular events	14.1	4.7	<0.001
New cardiogenic shock	7.6	1.9	<0.001
New heart failure	5.4	1.8	0.002
Coronary artery bypass grafting	3.4	1.1	0.04
Post-procedural myocardial infarction	10.8	1.6	<0.001

Values are median (interquartile range) or n (%). Adverse cardiovascular events are the composite of new cardiovascular shock and new heart failure before discharge.

CICAD = catheter-induced coronary artery dissection.

**Table 3:** Multivariable logistic regression analysis on in-hospital all-cause death and adverse cardiovascular events.

	All-cause death (n = 416)		Adverse cardiovascular events (n = 818)	
Variable	Adjusted OR (95% CI)	P value	Adjusted OR (95% CI)	P value
Age	1.05 (1.04–1.06)	<0.001	1.03 (1.03–1.04)	<0.001
Female gender	0.88 (0.67–1.15)	0.335	1.10 (0.92–1.32)	0.301
CICAD	3.87 (1.97–7.59)	<0.001	3.85 (2.42–6.11)	<0.001
Flow-recovered CICAD	1.55 (0.53–4.57)	0.425	2.26 (1.20–4.26)	<0.001
Flow-impaired CICAD	14.1 (0.56–35.4)	<0.001	10.9 (5.30–22.6)	<0.001
Cardiogenic shock at admission	38.4 (30.5–48.3)	<0.001	17.5 (14.6–21.1)	<0.001
Hemodialysis	4.40 (3.08–6.29)	<0.001	2.45 (1.85–3.24)	<0.001
Chronic obstructive pulmonary disease	1.44 (0.85–2.45)	0.179	1.39 (0.95–2.03)	0.090

CI = confidential interval; CICAD = catheter-induced coronary artery dissection; OR = odds ratio; PCI = percutaneous coronary intervention.

Of the CICAD patients, postprocedural flow impairment (TIMI flow ≤ 2) on the final angiogram was observed in 43 patients (23.2%). Patients with flow-limiting CICAD were associated with significantly increased rates of in-hospital all-cause mortality and adverse cardiovascular events than those without CICAD (18.6% vs 2.4%, p < 0.001; 30.2% vs 4.7%, p < 0.001, respectively). Patients with flow-recovered CICAD had higher rate of in-hospital adverse cardiovascular events than those without CICAD (9.2% vs 4.7%, p = 0.025), whereas in-hospital mortality was not significant (2.8% vs 2.4%, p = 0.58). A multivariate logistic regression analysis revealed that both flow-limiting (OR, 10.9; 95% CI, 5.30–22.6) and flow-recovered (OR, 2.26; 95% CI, 1.20–4.26) CICAD groups were at increased risk of in-hospital adverse events than the no-CICAD group after the adjustment for age, sex, cardiogenic shock at admission, maintenance dialysis, and chronic obstructive lung disease. In terms of in-hospital all-cause death, flow-impaired CICAD (OR, 14.1; 95% CI, 0.56–35.4) was at increased risk, while flow-recovered CICAD (OR, 1.55; 95% CI, 0.53–4.57) was not. (Table 3).

## Discussion

In this large-scale analysis of PCI in the contemporary era, the incidence and clinical impact of CICAD as a procedural complication was described. CICAD was observed in ~1% of all PCI, although the incidence of CICAD was decreasing over the study period. CICAD was associated with in-hospital mortality and adverse cardiovascular events. Furthermore, those with residual flow limitations were at a greater risk of postprocedural complications.

The overall incidence of CICAD was approximately half that of patients included in the previously published series [5,6]. This presumably reflects the advance in the technique applied and/or devices used to perform contemporary PCI despite increasing complexity of the target lesions. In the study period, the prevalence of complex PCI increased from 32.4% in 2009 to 43.1% in 2016. The use of second generation or newer DES was 47.3% in 2010 and increased to 86.8% in 2016. The prevalence of PCI with trans-radial approach was 20.8% in 2009 and gradually increased to 74.8% in 2016. Our dataset is derived from this “all-comer” consecutive registration system with outcome adjudications in which both patient and technique-related factors were assessed. Thus, although CICAD reporting was based on the procedure reports, the findings provide valuable and unique insight into the real-world occurrence of CICAD in the era of complex PCI. Differences in the CICAD rate may also reflect publication bias, as CICAD may be observed more frequently in specialist centers.

Importantly, in the current study, CICAD was clearly associated with in-hospital mortality and adverse cardiovascular events. In the pre-stent era, CICAD occurred in approximately 30% of all angioplasties [1–3]. Distal flow was recovered in approximately 40% of CICAD at final angiography, but patients with final flow limitations were at significantly higher risk of in-hospital complications, including abrupt closure, need for emergent coronary artery bypass grafting, and myocardial infarction [1]. In the first-generation DES era, although the incidence of CICAD had decreased to roughly 3% [4] and a normal distal flow was observed in about 80% of cases [5], a final flow-limiting dissection was associated with 1-month major cardiovascular events (composite of death, myocardial infarction, or target vessel revascularization). A similar result was observed in our study in that patients with flow-impaired CICAD were at a 3.3-fold increased risk of in-hospital adverse events compared to those without CICAD.

Our data also revealed that flow-recovered CICAD was also associated with an increased incidence of in-hospital adverse events. Few previous studies have focused on patients whose distal flow after CICAD was recovered by bailout PCI or medication since patients with preserved coronary flow after PCI were thought to have good clinical outcomes [1–3,5,21,22]. Biondi-Zoccai et al. reported that 49 patients who experienced non-obstructive CICAD at final angiography had a worse prognosis compared to those without CICAD (1-month composite of death, myocardial infarction, or target vessel revascularization: 12.2% vs 5.2%, p = 0.043) [5], consistent with our report. In terms of the angiographical evaluation of coronary flow, a recent study demonstrated that approximately 40% of patients with normalized TIMI flow after bailout PCI were estimated to have insufficient microvascular perfusion [23]. Although optimal distal flow was preserved after bailout PCI, the persistent insufficient microvascular flow caused by CICAD inflicts additional myocardial injury and necrosis, which may be associated with the increased risk of cardiovascular events [24].

Female sex and PCI of complex coronary anatomy were independent predictors of CICAD in our study. Higher rates of bleeding and vascular complications during and after PCI in women were previously reported [25–29]; however, the sex-based difference in the incidence of CICAD has not been clarified. Previous reports demonstrated that drastic changes in estrogen and progesterone levels in women were associated with plaque instabilities and endothelial cell dysfunction, which may increase the shear stress of the vessel wall and risk of dissection [30–32]. Moreover, women tend to have smaller body size and coronary arteries than men that are more susceptible to CICAD during PCI [28,33]. In terms of PCI of a complex coronary anatomy, previous studies demonstrated that a high rate of procedural complications in cases of complex PCI [8,9]. The studies conducted in the era of bare-metal stents and first-generation DES demonstrated that CTO (14.3% vs 5.2%, p < 0.001), calcification (30.0% vs 13.7%, p = 0.002), ACC/AHA lesion type B2 or C (44.2% vs 28.3%, p = 0.002) were observed at a higher rate in patients with final dissection [5], and severely calcified long and tortured lesions were associated with the increased risk of dissection [3,11]. Previous report demonstrated that sustained severe dissection occurred in 1.4% among 3622 PCIs to complex lesions (thrombus, calcification, bifurcation or CTO) [34]. As for calcification lesions, coronary dissections which required further stent deployment or prolonged balloon inflation occurred in 4.8% of PCI to severe calcificated lesions [35]. Although the definition of complex PCI and coronary dissection were different from the definitions in these reports, the incidence of severe CICAD in patients with complex PCI was 1.6% in our registry, which was similar to the previously reported incidence.

These results underscore the fact that an appropriate patient selection for PCI may be necessary for women with a complex coronary anatomy. Appropriate use of non-invasive imaging modalities (eg. coronary computed tomography angiogram) would be particularly important in these patients [36] and might be effective in minimizing (or avoiding) the risk of procedural complications. After her research is warranted to elucidate their risk evaluation methods and the indication of interventions to ensure the safety of PCI.

### Limitations

First, this was observational, which might carry inherent selection bias and unmeasured confounding factors, although the relevant measured confounders were analyzed in a multivariate analysis. This registry was established to evaluate the safety and effectiveness of PCI. Therefore, those who underwent diagnostic coronary angiography were not included. Moreover, we did not analyze the potential relationship between hospital or operator procedure volume and incidence of CICAD. In particular, there may be a relationship between an institution’s or operator’s procedure experience and the complications. Our registry only included the patients who underwent PCI at major teaching institution in Kanto area of Japan. To maximize data quality and minimize data loss, sophisticated clinical research coordinators collected more than 200 variables from each patient. Second, wire type, guiding catheter type, and stent/balloon inflation pressure were not recorded in this database. Third, NHLBI classification of coronary artery dissection type [37] was not recorded in this study. We could not identify the number of patients who had coronary dissection without any impairment in TIMI flow since the definition of CICAD in this registry was the coronary dissection which caused distal flow limitation (TIMI flow grade ≤ 2). However, we only recorded NHLBI type C–F dissections with flow limitation as CICAD since interventionist care is required.

## Conclusions

CICAD with flow limitation occurred in 1.1% of the study population, and about a quarter of these cases had persistent flow impairments. Female sex and complex PCI were the independent risk factors of CICAD. CICAD was associated with adverse in-hospital cardiovascular events regardless of final flow impairment status in this contemporary PCI era. Our data implied that the appropriate selection of PCI was necessary for women with complex lesions. The elective coronary bypass graft surgery should be considered if female patients present with complex coronary lesions with moderate to severe ischemia.
